# Comparison of Mirtazapine and Olanzapine on Nausea and Vomiting following Anthracycline-cyclophosphamide Chemotherapy Regimen in Patients with Breast Cancer

**DOI:** 10.22037/ijpr.2020.113955.14584

**Published:** 2020

**Authors:** Alimohammad Maleki, Mojtaba Ghadiyani, Jamshid Salamzadeh, Sina Salari, Seyedshahab Banihashem, Maria Tavakoli-Ardakani

**Affiliations:** a *Department of Clinical Pharmacy, School of Pharmacy, Shahid Beheshti University of Medical Sciences, Tehran, Iran. *; b *Department of Medical Oncology, Hematology and Bone Marrow Transplantation, Taleghani Hospital, Shahid Beheshti University of Medical Sciences, Tehran, Iran. *; c *Food Safety Research Center, School of Pharmacy, Shahid Beheshti University of Medical Sciences, Tehran, Iran. *; d *Department of Psychiatry, Taleghani Hospital, Shahid Beheshti University of Medical Sciences, Tehran, Iran. *; e *Pharmacuetical Sciences Research Center, Shahid Beheshti University of Medical Sciences, Tehran, Iran.*

**Keywords:** Mirtazapine, CINV, Complete response, Nausea, Chemotherapy

## Abstract

We evaluated and compared the efficacy and safety of mirtazapine (MTZ) with olanzapine (OLP) for preventing chemotherapy-induced nausea and vomiting (CINV) following anthracycline plus cyclophosphamide (AC) regimen. Eligible participants were chemotherapy-naive early-stage breast cancer patients who were scheduled to undergo adjuvant AC. The patients were randomized to take oral MTZ or OLP in combination with aprepitant (A), dexamethasone (D), and granisetron (G), (ADG). The endpoints included rates of complete response (CR), complete control (CC), total control (TC), and adverse events during the acute, delayed, and overall phases in the two cycles of chemotherapy. The influence of CINV on the quality of life (QoL) was evaluated on day 6 of chemotherapy. Of 82 patients, 60 were randomized. In the first cycle, CR rates in cycle 1 were 83.3% and 76.6% during the acute period, 80% and 86.6% during the delayed period, and 66.6% and 63.3% during the overall period, for the ADG-M and ADG-O, respectively. High efficacy of both groups was maintained over 2 cycles. More patients in the ADG-M group noted minimal or no impact of CINV on daily life in cycle 2 (89.7% *vs.* 67.9%; *p = *0.044). Incidence of somnolence and fatigue was more frequent with the olanzapine group. In this study, there was no substantial difference between mirtazapine and olanzapine in preventing CINV. Further large randomized trials are essential to demonstrate the anti-emetic effect of mirtazapine in chemotherapy.

## Introduction

Highly emetogenic chemotherapy (HEC) such as AC-based regimen without any antiemetic prophylaxis can cause nausea and vomiting (NV) in over 90% of the patients of breast cancer (1-3). Because NV following chemotherapy reduces patient’s QoL and compliance, and also causes a high economic burden on the health system, it is important to prevent NV following chemotherapy as much as possible (4, 5). Some clinical guidelines have already confirmed olanzapine as an effective therapeutic option in acute and delayed CINV prophylaxis (6-8). According to several studies in recent years, the American Society of Clinical Oncology (ASCO) and National Comprehensive Cancer Network (NCCN) guidelines recommended OLP as a first-choice option in combination with standard triplet regimen for patients receiving HEC as a new quadruple standard antiemetic regimen [Neurokinin-1 (NK-1) receptor antagonist+dexametasone+5-hydroxytryptamine 3 (5HT_3_) receptor antagonist+OLP] (9).

Chow *et al.* directed a meta-analysis to evaluate the efficacy of an olanzapine-based regimen in preventing early, delayed, and overall periods of CINV (10). They reported that no-nausea rates in the early, delayed, overall were 82.8%, 69.9%, and 66.9%, respectively. Furthermore, the rates of no emesis in the early, delayed, and overall periods were 85.9%, 77.3%, and 76.8%, respectively. Based on these data, OLP showed superior results in the delayed phase, which might be due to the low frequency of CINV during the acute phase.

Despite guideline-recommended quadruple-drug prophylaxis, delayed NV, especially nausea, can still have significant influences on patient’s outcomes and many patients still suffer loss of appetite with reduction of their oral and fluid intake during the delayed phase (11-15). Further, the short-term use of OLP in emesis management has been associated with drowsiness, fatigue, and disturbed sleep (10, 15-18). In some studies, OLP resulted in a more than 50% drowsiness rate and a 5% incidence of severe sedation (11, 18-20). Thus, other anti-nausea and emetic medicines are still required. 

Mirtazapine is a blocker agent at Histamine (H_1_), alfa_2_ adrenergic, 5HT_2C_, 5HT_2A_, and 5HT_3_ receptors (21). Previous studies have noted the effective role of MTZ in preventing emesis in cancer patients (21, 22-25). A recent phase ΙΙΙ CINV prevention study has shown that the addition of mirtazapine to triplet treatment has a sufficient and statistically meaningful benefit with adequate tolerance in cancer patients who have suffered delayed NV following the same earlier HEC (21). 

In addition to its antiemetic effects, mirtazapine has accelerating gastric emptying, appetite-stimulating, and sleep quality improvement or anxiolytic effects. Indeed, it could be considered a highly beneficial antiemetic agent for enhancing the overall QoL in post-chemotherapy (26-31).

We hypothesized that adding mirtazapine to the triplet regimen can further decrease the frequency of CINV and improve patientsʹ QoL compared to olanzapine. The primary purpose was to compare the efficacy and safety of MTZ with OLP (both in combination with triplet regimen) for the prophylaxis of CINV in Iranian patients with early-stage breast cancer during the first 2 cycles of doxorubicin/cyclophosphamide chemotherapy.

## Experimental

*Trial design*

This prospective, randomized, parallel-arm, phase ΙΙΙ study was performed at two clinics in Iran from December 15, 2019, to May 14, 2020. The study was recorded at the Iranian registry of clinical trials (Identifier: IRCT20100127003210N19) and submitted by the Medical Research Ethics Committee (MREC) before any data collection procedures. All of the patients provided written informed consent to participate in the trial. 

*Participants*

Eligible participants were females aged 18-65 years with early-stage breast cancer who had not received previous chemotherapy if they were planned to undergo adjuvant AC therapy (doxorubicin ≥50 mg/m^2^and cyclophosphamide ≥500 mg/m^2^) for the first two consecutive cycles of chemotherapy.

Exclusion criteria were hypersensitivity to aprepitant, dexametasone, granisetron, MTZ, and OLP; known history of active gastroduodenal ulcer, cardiac arrhythmia, CNS disease, glaucoma, myocardial infarction, peptic ulcer, serious emotional or mental disorders, uncontrolled diabetes mellitus, congestive heart failure; concurrent use of any drug with class X and D interaction (*e.g.*, antidepressants and antipsychotics) with the drugs studied; concurrent use of systemic steroid or antiemetic drug and other drugs with inducer or inhibitory effects on NV; an aspartate or alanine aminotransferase level more than 3 times the upper limit of normal; a serum creatinine level more than 2.0 mg per deciliter; an absolute neutrophil count (ANC) count less than 1500 per cubic millimeter.

*Randomization and masking*

A randomized block plan was employed to ensure a proportional allocation to each trial arm. A random assignment schedule was created by a pharmacist who was otherwise not involved in the study. Age (<55 years or ≥55 years) was used as a factor of allocation adjustment. After stratification, eligible participants were randomly allocated (1:1) to receive ADG-M or ADG-O regimen. To ensure the double-blinding of the trial, medication assignments at two contributing clinics were conducted by independent pharmacists not involved in the allocation schedule. The medical team, principal investigator, outcome assessor, and data analyzer were blinded to the treatment assignment.

*Interventions*

All participants received AC-based chemotherapy in an outpatient setting which consisted of doxorubicin ≥50 mg/m^2^ plus cyclophosphamide ≥500 mg/m^2^. The patients were evaluated for the first 2 cycles of chemotherapy. On day 1, 30-60 min before chemotherapy, the patients were randomized to take aprepitant, dexamethasone, granisetron, and mirtazapine (ADG-M) or aprepitant, dexamethasone, granisetron, and olanzapine (ADG-O).

All of the patients received 125 mg of oral aprepitant on day 1 and then 80 mg on days 2-3, 12 mg of IV dexametasone and 1mg of IV granisetron on day 1. In the ADG-M group, mirtazapine was orally administered at a dose of 15 mg on days 1-4 after chemotherapy. In the ADG-O group, olanzapine was orally administered at a dose of 10 mg on days 1-4 after chemotherapy. OLP 5 mg was used in patients aged 60 years and older.

The participants were allowed to receive rescue treatment (other antiemetic therapy) for nausea or vomiting throughout the study based on clinical circumstances. Consistent with the recommendation by NCCN and ASCO in the AC setting, olanzapine and aprepitant were given on days 1-4 and 1-3, respectively. Also, dexametasone was given on day 1 only (9). MTZ and OLP drugs had been made by Tadbir Kala Jam and Sobhan Darou pharmaceutical companies, respectively.

*Evaluation methods and study visits*

Before the chemotherapy, all relevant demographics and clinical information were recorded by a trained research pharmacist. The participants were instructed to report everyday severity of NV during the early (0-24 h after chemotherapy), delayed (24-120 hours after chemotherapy), and overall (0-120 h after chemotherapy) periods according to the National Cancer Institute’s Common Terminology Criteria for Adverse events (NCI-CTCAE), version 5.0 (32).

All participants were also asked to keep a diary card for reporting any emetic episodes, nausea, usage of rescue medications, and details of adverse events (AEs) every 24 h for the day before and 5 days following the first two chemotherapy cycles. Adverse events (somnolence, fatigue, constipation, headache, insomnia, dry mouth, dizziness, diarrhea, and loss of appetite) were assessed every 24 h by a pharmacist, according to the NCI-CTCAE version 5.0. Events of vomiting and retching were both considered emetic cases. 

All patients were in an outpatient setting during the 5 days of observation. The pharmacist contacted individual patients by telephone on days 2 through 6, to consult them about possible AEs and remember them to fill in the diary forms, to take the study medicines as advised, and to assist them to fulfill the Functional Living Index-Emesis (FLIE) questionnaire. The patients were assessed for the first 2 courses of AC chemotherapy.

*Outcomes*

The primary outcome in the study was complete response (CR; no vomiting, and no use of rescue medication) for the acute period (0-24 h after chemotherapy). The secondary endpoints were CR in the delayed (24-120 h after chemotherapy) and overall period (0-120 h after chemotherapy), complete control (CC; no vomiting, no use of rescue therapy, and no significant nausea), total control (TC; no vomiting, no rescue therapy, and no nausea), no nausea (response of 0 on NCI grading for nausea) , no significant nausea (NCI grading for nausea <3) , no vomiting (response of 0 on NCI grading for vomiting), no rescue therapy, and safety profile (AEs) during the three-time frames. A significant nausea was considered as grade of nausea ≥ 3 based on NCI toxicity grading. Further, the influence of CINV was assessed on QoL on day 6, following AC chemotherapy of cycles 1 and 2. The frequency of CINV was evaluated simply on a “yes” or “no” explanation.

Also, NCI-CTCAE version 5.0 was used to assess patient AEs and CINV experiences, based on the severity level (0 to 4; 4 = most severe) of adverse events during the first five days after chemotherapy. For safety assessment, incidences of adverse events appearing in more than three percent of participants would be summarized by the treatment arm. Compliance of therapy was checked with the amount of drugs taken and remained each day and at the end of the treatment. Participants were permitted to use rescue therapy (lorazepam tablet) throughout the trial for breakthrough CINV according to NCCN guidelines. The QoL was evaluated via the reliable and validated Iranian interpretation of the self-reported FLIE questionnaire by individual patients. It includes 18 questions which mainly measure the effects of CINV on the ability to maintain usual recreation or leisure activities, daily functioning, social and enthusiastic capacities, and the ability to take pleasure in meals and drinks. Each response is based on a 1-7 degrees visual analog scale (VAS) with “1 degree” relating to “not in any way” and “7 degrees” relating to “a lot”. “No influence on daily living” was suggested as average overall FLIE score <36 (33-35).

*Statistical analysis*

To have 80% power to the difference in the acute CR ratings between the two treatment regimens with a two-sided 5% level test, the participants’ number was a total of 54 patients. Assuming that approximately 10% of participants would withdraw or drop out, a target sample size of 30 participants per study arm (a total of 60 patients) was determined. A Chi-square test was utilized to compare the primary and secondary outcomes as well as the influence of CINV on daily living. Mann-Whitney u test was applied to make therapy comparisons concerning the frequency of adverse effects.

## Results

**Figure 1:**
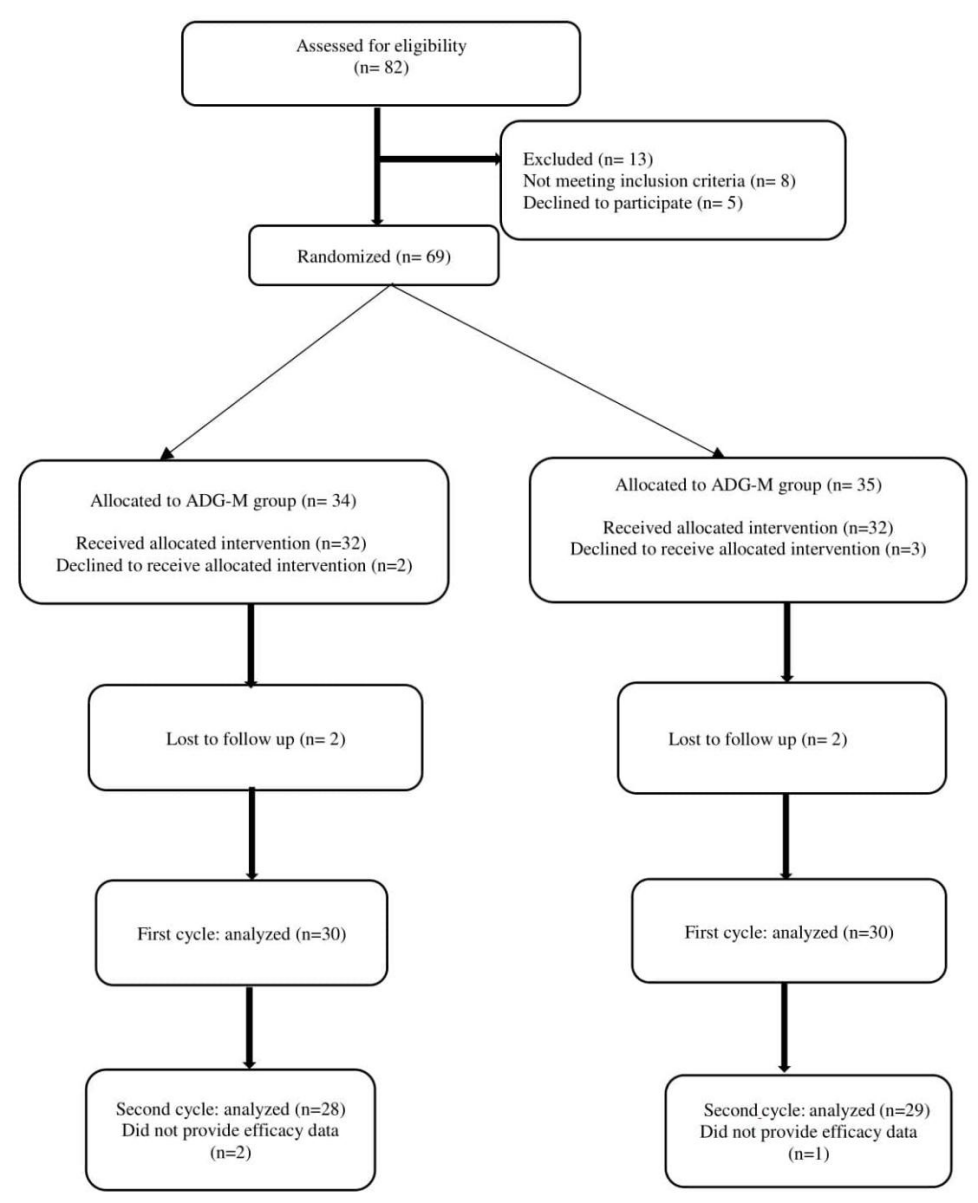
Consort flow chart; ADG-M, Aprepitant, Dexametasone, Granisetron, and Mirtazapine; ADG-O, Aprepitant, Dexametasone, Granisetron, and Olanzapine

Between December 15, 2019, and May 14, 2020, 82 patients were enrolled in the ADG-M and ADG-O groups. Figure 1 presents a consort chart of allocation and randomization of the 69 participants treated with AC-based chemotherapy regimens. Of the 69 randomized patients, five patients declined to receive an antiemetic regimen, 2 patients were lost to follow up in response to lack of CINV data, and 3 patients did not complete the intervention. Accordingly, 60 patients (30 in ADG-M and 30 in the ADG-O) were involved in the final data analysis. In the second cycle, 57 patients (29 in the ADG-M group and 28 in the ADG-O group) were involved in the effectiveness and safety analysis. All eligible patients were given quadrupled-combination therapy as scheduled. The adherence rates of patients throughout the first cycle of AC chemotherapy were 100%, but in the second cycle, 1 patient in the ADG-M arm and 2 patients in the ADG-O arm did not provide efficacy data in the delayed periods.

The patient characteristics were well balanced between ADG-M and ADG-O. Almost 70% of the patients in the two groups were young (<55). No apparent differences in baseline characteristics were detected between the two groups (Table 1).

*Efficacy*

**Figure 2:**
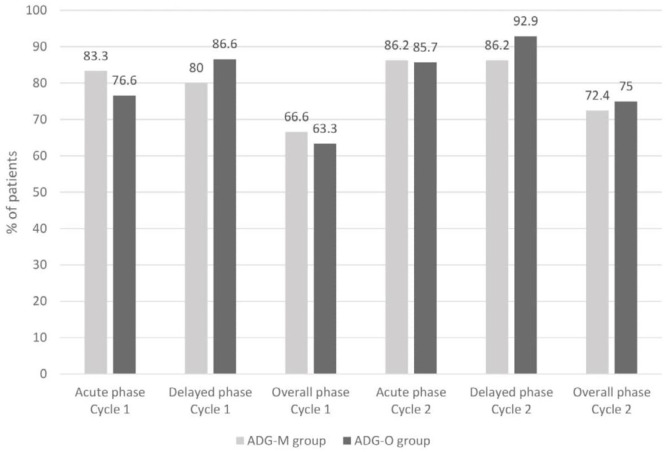
*Percentage of patients achieving complete response of CINV patients receiving doxorubicin/cyclophosphamide regimen in cycle 1 and 2 of chemotherapy; ADGM, aprepitant, dexamethasone, granisetron, mirtazapine; ADGO, aprepitant, dexamethasone, granisetron, olanzapine*

For the primary efficacy comparison, Figure 2 displays the percentage of patients in each study arm who had CR in the first two cycles of AC chemotherapy. At the first cycle, there were no substantial differences in the complete response between the ADG-M arm and the ADG-O arm for the early (*p = *0.51), delayed (*p = *0.48), and overall (*p = *0.78) phases. At the second cycle, better results were observed with CR for both groups. There were no substantial differences in the complete response between the ADG-M arm and the ADG-O arm for the acute (*p = *1.00), delayed (*p = *0.67), and overall (*p = *0.59) phases.

The secondary outcomes of CINV between the two groups over 2 cycles of chemotherapy are listed in Table 2. There was no statistically meaningful difference between the early and delayed phases in terms of CC frequency; TC rate; and rates for no vomiting, no rescue therapy, and no significant nausea. In the overall phase of the first cycle, 9 (30%) of 30 patients in the ADG-M group and 7 (23.3%) of 30 in the ADG-O group and then in the second cycle, 4 (13.8%) of 29 participants in the ADG-M arm and 6 (21.4%) of 28 patients in the ADG-O arm had nausea or vomiting, who required additional treatment with rescue therapy. 

*Safety*

Adverse drug events were shown in two groups in Table 3. In cycles 1 and 2, there were no grade 3 AEs in the ADG-M arm. The frequency of somnolence and fatigue was considerably lower in the ADG-M group than in the ADG-O group. In the ADG-O group, two patients (6.7%) in cycle 1 and one patient (3.6%) in cycle 2 had an NCI grade 3 (severe) somnolence, and 2 patients in cycle 1 (6.7%) and 2 patients (7.1%) in cycle 2 had fatigue NCI toxicity grade 3 (severe). There were no grade 4 adverse events in the two treatment arms. There was no meaningful difference in other AEs (insomnia, dry mouth, loss of appetite, constipation, headache, dizziness, and diarrhea). Fatigue grade 3 was not attributed to MTZ or OLP by the medical team. These adverse effects did not influence the patients´ daily living nor did them need medications.

*The assessment of FLIE*

**Figure 3:**
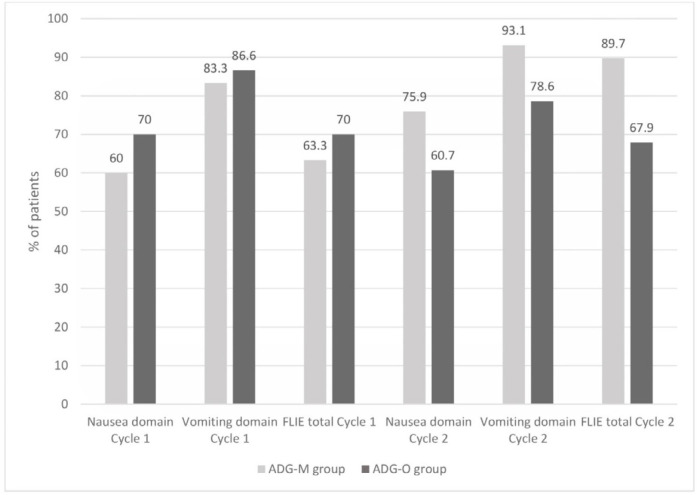
*Percentage of patients with no impact on daily living according to Functional Living-Index Emesis (FLIE); ADGM, aprepitant, dexamethasone, granisetron, mirtazapine; ADGO, aprepitant, dexamethasone, granisetron, olanzapine*

The percentage of participants with no influence of CINV on daily living on day 6 is shown in Figure 3. In cycle 1, there were no meaningful differences in the mean overall score of FLIE. However, in cycle 2, the percentage of the patients with no influence on daily living were greater in the ADG-M arm than in the ADG-O arm (89.7% *vs. *67.9%, *p =* 0.044).

## Discussion

This trial, as far as we could know, was the first randomized trial to compare the effectiveness and safety of MTZ with OLP for preventing CINV following AC therapy in breast cancer patients. Meanwhile, this is the first trial of mirtazapine in a homogenous group of Iranian breast cancer patients undergoing a uniform protocol of adjuvant doxorubicin/cyclophosphamide chemotherapy. Mirtazapine combined with the triplet antiemetic regimen was a beneficial alternative at preventing acute and delayed CINV in participants undergoing AC chemotherapy compared with olanzapine. There was no meaningful difference identified in the control of CINV using a mirtazapine-based quadrupled therapy when compared with the olanzapine-based quadrupled regimen in Iranian breast cancer participants undergoing AC-based HEC. 

Mirtazapine, as with olanzapine, binds with high affinity to several receptors involved in the CINV pathways including serotonin (5-HT_2A_, 5-HT_2C_, 5- HT_3_, and 5-HT_6_), histamine (H_1_), α_1_ adrenergic, and acetylcholine-muscarine receptors (11, 36). For these reasons, the usage of mirtazapine as with olanzapine, combined with triplet regimen is believed to demonstrate an antagonistic role on a large portion of the receptors associated with CINV.

The two antiemetic regimens were comparable in CR, CC, TC rate in the early, delayed, and overall periods. The efficacy findings of CR and CC with the mirtazapine-based regimen seen in the present trial were consistent with those in a previous clinical trial by Cao *et al.* (21). They evaluated the efficacy of a four-drug combination including mirtazapine in controlling delayed NV following HEC. The CR rates in the first cycle were significantly higher in the mirtazapine study arm compared with triplet regimen: 78.3% vs. 49.0%, *P = *0.003, in the late period, and 58.7% vs. 34.7%, *p = *0.019, during all phases. The frequencies of CC were also quite higher with mirtazapine: in the first cycle, 76.1% *vs.* 49.0%, *p = *0.006, in the late period and 56.5% *vs.* 32.7%, *p = *0.019, during all periods; This study found that the addition of mirtazapine improves CINV which may be sustained over several courses of chemotherapy. 

Cao *et al.* ʹs trial and our study demonstrated the maintenance of effectiveness of mirtazapine-based quadrupled therapy in the patients undergoing repeated courses of chemotherapy. The difference between our study and Cao *et al.* was the comparable arm with mirtazapine. The lack of difference in the effectiveness between mirtazapine and olanzapine is not limited to the prevention of early emesis; rather it has also been found in preventing delayed NV. This isn’t unexpected, as one of the most notable components essential for good prevention from the late NV is achieving good management of early NV, as happened in our trial with the two study arms (21, 37 and 38). The addition of mirtazapine to the triplet therapy showed a favorable to the antiemetic effect throughout the three phases, especially in the acute phase following AC chemotherapy. Such outcomes can partly be interpreted by the pharmacokinetics of the drug. The maximum of plasma concentration is achieved 2 h after a single dose of mirtazapine and its elimination half-life can be as long as 20–40 h. The fast and long outcomes of mirtazapine can, therefore, protect against nausea and vomiting following AC chemotherapy and provide sustained control of CINV, as observed in our patients (39, 40).

For participants receiving AC observed in this trial, the high level of CR, CC, and TC in the early phase was mostly reasonable a valuable element in preventing delayed NV. The significance of the control of early CINV in controlling delayed NV has been noted in other trials (38, 41-43). Thus, patients who had no emesis in the early phase were far more likely to remain emesis-free in the late phase if they received the ADG-M or ADG-O regimen. This randomized study showed that the efficacy of prophylaxis against delayed NV following HEC is strongly influenced by the experiences of these adverse events during the acute phase post-chemotherapy.

The effect of mirtazapine and olanzapine, 5HT_3_–receptor blockers, in previous studies on the control of emesis indicates that serotonin may play an important function in the pathogenesis of emesis (44). HEC (*e.g.*, AC) is proposed to induce serotonin release, thereby provoking 5HT_3 _receptors and contributing to CINV. Serotonin blockers are expected to be useful in early emesis following chemotherapy as serotonin is released rapidly from the GI tract within the initial 24 h (45, 46).

In addition to the 5HT_3_ blocker properties, mirtazapine can affect its anti-emetic properties in several different possible ways. It has a highly antihistaminic, anxiolytic, and antidepressant effect by stimulating the 5HT_2_ or 5HT_1A_, and the interactions between neurokinin-1 (NK-1), serotonin, and adrenaline receptors have also been shown. Mirtazapine might exert its anti-NV influence indirectly by inhibiting the NK-1 receptors’ action and the neuronal excitability by modulating the openings of calcium channels and reducing neurotransmitter release. Further, there is cross-talk between 5HT_3_ receptors and voltage-gated Ca^2+^ channels. Finally, mirtazapine has gastric pro-kinetic and appetite improving properties (40).

 If NV following chemotherapy is beneficially prevented in the first course of chemotherapy, the patient is the potential to have useful control during subsequent courses of identical chemotherapy. On the other hand, if the patient has poor management of NV in the first course of chemotherapy, it may be more difficult to prevent NV in subsequent courses, and anticipatory NV may develope (17). This is consistent with the use of mirtazapine in this study.

 We observed that the frequency of CR in the present study was also higher than that in previous trials reporting the effectiveness of the triplet regimen in the patients receiving HEC (11, 33, 44, 47 and 48). In a large randomized phase 3 trial published by Navari *et al.*, which assessed participants undergoing different HEC treatments, the CR rates also significantly increased with the olanzapine-based quadrupled regimen compared with the triplet regimen in the early (86% *vs.* 65%), late (67% *vs.* 52%) and overall (64% *vs.* 41%) periods (11). However, in our trial, the CR rates for mirtazapine group in cycle 2 were 86.2%, 86.2%, and 72.4% in the early, delayed, and overall periods, respectively.

 Although the two groups did not differ statistically for no nausea, no significant nausea, and no vomiting, the overall frequencies for these outcomes were consistently numerically higher in the mirtazapine arm in cycle 2 of chemotheapy. The higher response rates of these endpoints in the mirtazapine group, defined in the context of a reasonably less frequent need for rescue treatment, suggest that mirtazapine provided some benefit against nausea as well as emesis.

 Olanzapine has been shown in earlier trials to be an effective drug at preventing delayed CINV (18, 43, and 48) which is congruent with our results in the olanzapine arm that CR and no nausea rates were numerically higher in the delayed period compared with the early phase. Conversely, these outcomes were numerically higher in the acute phase in the mirtazapine study arm. 

As observed in previous trials, the mirtazapine therapy was well tolerated (21, 22, 30, 39, and 49). The primary AEs were somnolence and fatigue, dry mouth, and constipation. Initial studies evaluating olanzapine use for NV following HEC indicated a tendency toward more somnolence and fatigue with the olanzapine regimen than with the control group (11, 16, 20, 33 and 50). In our study, the only substantial differences between the study arms were higher incidences of somnolence (*p = *0.04) and fatigue (*p = *0.02) with the olanzapine regimen. In comparison with mirtazapine, there were 5% of patients with severe sedation in the olanzapine group. Thus, this is consistent with the trial by Navari *et al.* while inconsistent with the previous studies which have reported no severe sedation with olanzapine (11, 17, 18, 43, 51 and 52). In some earlier trials, the incidence of olanzapine-induced somnolence was more than 50% at a dose of 10 mg/day (11, 16, 20 and 33). Thus, further studies such as randomized trial by Hashimoto *et al.* may be needed to determine the safety and efficacy of olanzapine 5 mg (53). 

 In contrary to the ASCO guideline, the NCCN guideline still recommends dexamethasone for delayed NV following chemotherapy (9). In some patients, the uses of dexamethasone need to be weighed against the increased risk of potential adverse events (53). In this trial, the benefits of mirtazapine and olanzapine in managing delayed NV following chemotherapy were achieved without requiring dexamethasone within the 2-3 days post-chemotherapy of AC, potentially eliminating the adverse events of dexamethasone including insomnia (45%), agitation (27%), GI discomfort (27%), skin rash (15%), hyperglycemia, and immunosuppression (21, 48 and 54).

The FLIE questionnaire, in this study, was used to evaluate the potential of beneficial anti-emetic regimen to prevent an adverse impact of CINV on patientsʹ daily lives (34, 55). Analysis of the influence on daily living indicated that while there were no differences in FLIE scores between the two study arms in cycle 1 AC, there was a significantly better QoL (lower FLIE scores) based on the mean total score (mean score [SD] for mirtazapine arm *vs. *olanzapine arm: 25.6 [10.6] *vs.* 30.1 [14.2] respectively, *p = *0.044) among participants in the mirtazapine arm in cycle 2 on Day 6 following AC chemotherapy. Thus, mirtazapine appears to have apparent usefulness for CINV, with no evident adverse better impact on QOL. We conclude that nausea had a stronger negative impact on QoL than vomiting. Accordingly, we noted that use of mirtazapine-based quadrupled regimen might enhance patientsʹ QoL to some extent in cycle 2 of chemotherapy.

There were several limitations to the current study. Initially, the number of participants was relatively small, though the design was a randomized, double-blinded trial. Secondly, the effect of more than two courses of chemotherapy was not assessed. Further, we analyzed only one dose level of mirtazapine. Lower or higher doses may influence adverse events, efficacy, or both. The optimal dose of mirtazapine expected to control CINV is uncertain. Thus, for further evaluation, a larger-scale, prospective, randomized controlled phase 3 trial is essential to demonstrate the effect of mirtazapine in patients undergoing HEC.

On the other hand, the study was double-blinded and all of the participants were homogenous groups of early-stage breast cancer participants of Iranian ethnicity who were scheduled for a uniform adjuvant doxorubicin/cyclophosphamide treatment. Further, the patient characteristics (baseline risk factors) were well balanced between regimens ADG-M and ADG-O. There were no meaningful differences between the two arms in participantsʹ eligibility, study assessments, standard triplet therapy, AC chemotherapy regimen, age, and history of emesis with pregnancy. 

**Table 1:** *Baseline characteristics*

**Characteristic**	**ADG-M (N = 30)**	**ADG-O (N = 30)**	***P***
Age (years)			0.73
Range	30-63	29-63	
Mean	47	46	
SD	10	11	
Stage of cancer, n (%)			0.94
I	2 (6.6)	1 (3.3)	
II	18 (60)	20 (66.6)	
III	10 (33.3)	9 (30)	
ECOG PS, n (%)			0.54
0	25 (83.3)	23 (76.6)	
1	5 (16.6)	7 (23.3)	
History of motion sickness, n (%)	5 (16.6)	7 (23.3)	0.52
History of morning sickness, n (%)	9 (30)	8 (26.6)	0.77
Alcohol intake history, n (%)	1 (3.3)	1 (3.3)	1.00

ECOG PS: Eastern Cooperative Oncology Group performance status; SD: Standard Deviation; ADG-M: Aprepitant, Dexametasone, Granisetron, and Mirtazapine; ADG-O: Aprepitant, Dexametasone, Granisetron, and Olanzapine

**Table 2:** *Percentage of patients with secondary emesis outcomes in the three-time frames during cycle 1 and 2 of AC chemotherapy*

**Groups**	**Acute (>0-24 h)**				**Delayed phase (>24-120 h)**			**Overall phase (0-120 h)**		
	**ADG-M (%)**	**ADG-O (%)**		***P***	**ADG-M (%)**	**ADG-O (%)**	***P***	**ADG-M (%)**	**ADG-O (%)**	***P***
Cycle 1	76.6	70.0	0.56		63.3	70.0	0.58	56.6	63.3	0.60
Cycle 2	86.2	71.4	0.17		69.0	78.5	0.41	62.0	53.5	0.60
Cycle 1	80.0	76.6	0.75		66.6	76.6	0.39	60.0	66.6	0.59
Cycle 2	89.7	85.7	0.71		82.8	82.1	0.95	75.9	67.9	0.50
Cycle 1	86.6	86.6	1.00		96.6	90.0	0.61	83.3	76.6	0.52
Cycle 2	89.7	85.8	0.71		93.1	92.9	1.00	82.8	78.6	0.69
Cycle 1	86.6	86.6	1.00		80.0	90.0	0.47	70.0	76.6	0.56
Cycle 2	93.1	85.7	0.42		93.1	92.9	1.00	86.2	78.6	0.50
Cycle 1	76.6	73.3	0.76		63.3	66.6	0.78	53.3	53.3	1.00
Cycle 2	79.3	75.0	0.69		75.9	75.0	0.94	62.0	53.6	0.51
Cycle 1	73.3	66.6	0.57		60.0	60.0	1.00	50.0	50.0	1.00
Cycle 2	79.3	67.9	0.32		62.0	71.4	0.45	55.2	50.0	0.69

ADGM, Aprepitant, Dexamethasone, Granisetron, Mirtazapine; ADGO, Aprepitant, Dexamethasone, Granisetron, Olanzapine.

**Table 3:** *Adverse events*

**Severity Somnolence**	**ADG-M group, n (%)**				**ADG-O group, n (%)**				
	**Grade 1**	**Grade 2**	**Grade 3**		**Grade 1**	**Grade 2**	**Grade 3**		***P***

Cycle 1	8 (26.7)	2 (6.7)	0 (0.0)		14 (46.7)	2 (6.7)	2 (6.7)		0.04
Cycle 2	5 (17.2)	2 (6.9)	0 (0.0)		13 (46.4)	1 (3.6)	1 (3.6)		0.03
**Fatigue**									
Cycle 1	8 (26.7)	2 (6.7)	0 (0.0)		13 (46.4)	4 (13.3)	2 (7.1)		0.02
Cycle 2	7 (24.1)	0 (0.0)	0 (0.0)		9 (32.1)	5 (17.9)	2 (7.1)		0.01
**Dry Mouth**									
Cycle 1	10 (33.3)	3 (10.0)	0 (0.0)		8 (26.7)	2 (6.7)	0 (0.0)		0.42
Cycle 2	9 (31.0)	1 (3.4)	0 (0.0)		12 (42.9)	0 (0.0)	0 (0.0)		0.60
**Constipation**									
Cycle 1	10 (33.3)	1 (3.3)	0 (0.0)		8 (26.7)	0 (0.0)	0 (0.0)		0.37
Cycle 2	8 (27.6)	1 (3.4)	0 (0.0)		6 (21.4)	2 (7.1)	0 (0.0)		0.92
**Loss of appetite**									
Cycle 1	1 (3.3)	12 (40.0)	0 (0.0)		1 (3.3)	10 (33.3)	0 (0.0)		0.59
Cycle 2	4 (13.8)	7 (24.1)	0 (0.0)		4 (14.3)	9 (32.1)	0 (0.0)		0.49
**Headache**									
Cycle 1	6 (20.0)	0 (0.0)	0 (0.0)		8 (26.7)	1 ( 3.3)	0 (0.0)		0.35
Cycle 2	4 (13.8)	1 (3.4)	0 (0.0)		8 (28.6)	1 (3.6)	0 (0.0)		0.18
**Dizziness**									
Cycle 1	3 (10.0)	0 (0.0)	0 (0.0)		2 (6.7)	0 (0.0)	0 (0.0)		0.64
Cycle 2	4 (13.8)	1 (3.4)	0 (0.0)		7 (25.0)	1 (3.6)	0 (0.0)		0.33
**Insomnia**									
Cycle 1	2 (6.7)	1 (3.3)	0 (0.0)		5 (16.7)	1 (3.3)	0 (0.0)		0.30
Cycle 2	2 (6.9)	2 (6.9)	0 (0.0)		6 (21.4)	2 (7.1)	0 (0.0)		0.21
**Diarrhea**									
Cycle 1	2 (6.7)	0 (0.0)	0 (0.0)		1 (3.3)	1 (3.3)	0 (0.0)		0.97
Cycle 2	2 (6.9)	1 (3.4)	0 (0.0)		2 (7.1)	0 (0.0)	0 (0.0)		0.65

ADGM, Aprepitant, Dexamethasone, Granisetron, Mirtazapine; ADGO, Aprepitant, Dexamethasone, Granisetron, Olanzapine.

## Conclusion

This randomized study revealed that mirtazapine, given at a dose of 15 mg, was not inferior to olanzapine in preventing nausea and vomiting following doxorubicin/cyclophosphamide chemotherapy. There was no substantial difference between mirtazapine and olanzapine in preventing nausea and vomiting with AC regimen in breast cancer patients. There were no significant differences in adverse effects of two drugs except somnolence and fatigue that are more in olanzapine group. Nevertheless, for further evaluation, a larger-scale, prospective, and randomized controlled trial is essential to demonstrate the effect of mirtazapine in patients undergoing HEC.
